# HSF1 and HSF3 cooperatively regulate the heat shock response in lizards

**DOI:** 10.1371/journal.pone.0180776

**Published:** 2017-07-07

**Authors:** Ryosuke Takii, Mitsuaki Fujimoto, Yuki Matsuura, Fangxu Wu, Namiko Oshibe, Eiichi Takaki, Arpit Katiyar, Hiroshi Akashi, Takashi Makino, Masakado Kawata, Akira Nakai

**Affiliations:** 1 Departments of Biochemistry and Molecular Biology, Yamaguchi University School of Medicine, Minami-Kogushi, Ube, Japan; 2 Department of Ecology and Evolutionary Biology, Graduate School of Life Sciences, Tohoku University, Sendai, Japan; Fred Hutchinson Cancer Research Center, UNITED STATES

## Abstract

Cells cope with temperature elevations, which cause protein misfolding, by expressing heat shock proteins (HSPs). This adaptive response is called the heat shock response (HSR), and it is regulated mainly by heat shock transcription factor (HSF). Among the four HSF family members in vertebrates, HSF1 is a master regulator of *HSP* expression during proteotoxic stress including heat shock in mammals, whereas HSF3 is required for the HSR in birds. To examine whether only one of the HSF family members possesses the potential to induce the HSR in vertebrate animals, we isolated cDNA clones encoding lizard and frog *HSF* genes. The reconstructed phylogenetic tree of vertebrate HSFs demonstrated that HSF3 in one species is unrelated with that in other species. We found that the DNA-binding activity of both HSF1 and HSF3 in lizard and frog cells was induced in response to heat shock. Unexpectedly, overexpression of lizard and frog HSF3 as well as HSF1 induced HSP70 expression in mouse cells during heat shock, indicating that the two factors have the potential to induce the HSR. Furthermore, knockdown of either HSF3 or HSF1 markedly reduced HSP70 induction in lizard cells and resistance to heat shock. These results demonstrated that HSF1 and HSF3 cooperatively regulate the HSR at least in lizards, and suggest complex mechanisms of the HSR in lizards as well as frogs.

## Introduction

All living cells must maintain the appropriate conformations and physiological concentrations of proteins, which is known as protein homeostasis or proteostasis, by keeping a balance between the synthesis, folding, and clearance of individual proteins [1–3]. To adapt to temperature elevations, which cause protein unfolding and misfolding, cells have evolved sophisticated mechanisms that adjust proteostasis capacity or buffering capacity against protein misfolding [4]. One such evolutionally conserved mechanism is the heat shock response (HSR), which is characterized by the induction of heat shock proteins (HSPs) or chaperones, and facilitates proper folding of cellular proteins [5]. The HSR is mainly regulated at the level of transcription by the widely conserved transcription factor heat shock factor (HSF), which binds to the heat shock response element (HSE) in eukaryotes [6–8].

In contrast to the single *HSF* gene in yeast, worm, and fruit fly, four *HSF* genes (*HSF1*, *HSF2*, *HSF3*, and *HSF4*) have been isolated in vertebrates, including chicken, mouse, and human [9, 10]. Among vertebrate HSFs, HSF1 is a master regulator of *HSP* expression during proteotoxic stresses, including heat shock, in mammals. HSF1 mostly remains as an inert monomer in unstressed conditions. In response to heat shock, it is converted to an active trimer that binds to HSEs in *HSP* promoters, and robustly induces the transcription of these genes [11, 12]. The induction of HSPs is associated with increased proteostasis capacity and resistance to cell death. Mammalian HSF2, HSF3, and HSF4 are also involved in the maintenence of proteostasis capacity by regulating the expression of *non-HSP* genes such as *αB-* and *γ-crystallins* and *PDZK3* [13–16]. However, they are dispensable for the induction of *HSP* expression during proteotoxic stresses [14, 17, 18], although they modify it to some extent [19, 20].

Of particular note, chicken HSF1 is dispensable for stress-induced *HSP* expression, indicating that it is functionally different from mammalian HSF1. Instead, chicken HSF3 (cHSF3) is required for the induction of *HSP* expression [21–23]. cHSF3 remains as an inert dimer in unstressed conditions, and forms an active trimer in response to heat shock [24]. Thus, cHSF3 has unique properties and plays a pivotal role in the regulation of the chicken HSR. However, the potential to induce *HSP* expression had been lost in mouse HSF3, and the human *HSF3* gene is a pseudogene [14]. Thus, the indispensable role of HSF3 has so far been considered to be specific in birds.

Recently, whole genomic sequences of a variety of animal species have been determined and released to the public. It was revealed that all four *HSF* genes exist in vertebrate species, including in reptiles and amphibians [10]. To uncover roles of HSFs in the HSR in vertebrates, we isolated complementary DNAs of lizard and frog HSF family members and examined their potential to induce *HSP* expression during heat shock. Unexpectedly, we found that both HSF1 and HSF3 have the potential to induce *HSP* expression in lizards and frogs.

## Materials and methods

### Cell cultures and treatments

Immortalized wild-type (stock #10) or HSF1-/- (stock #4) mouse embryonic fibroblasts (MEFs) [25], human HEK293 cells, and chicken DF-1 fibroblast (ATCC CRL-12203) [26] cell lines were maintained at 37^°^C in 5% CO_2_ in Dulbecco’s modified Eagle’s medium (DMEM; Gibco/Life Technologies) supplemented with 10% fetal bovine serum (FBS) (Sigma Life Science). Lizard gekko lung-1 (GL-1) epithelial cells isolated from *Gekko gecko* (ATCC CCL-111) [27] were maintained at 30^°^C in 5% CO_2_ in Ham's F-12K medium (Gibco/Life Technologies) supplemented with 15% FBS. Western clawed frog epithelial-like Speedy cells derived from *Xenopus tropicalis* (or *Silurana tropicalis*) tadpole hindlimbs [28] were provided by Dr. Nicolas Pollet (Institute of Systems and Synthetic Biology, Genopole, CNRS, France), and maintained at 28^°^C in Leibovitz's L-15 Medium (Sigma Life Science)/double distilled water (2:1) supplemented with 10% FBS. To induce heat shock in the cultured cells, the culture dishes were sealed with parafilm and submerged in a water bath at indicated heat shock temperature.

### Care and treatment of lizards and frogs

Adult female brown anole lizards, *Anolis sagrei*, were collected in Havana city, Cuba during 2009 to 2011 (for RNA isolation), and transported alive to Tohoku University, Japan as described previously [29]. Briefly, we collected *A*. *sagrei* in Havana City, which was not a protected area, and it is not an endangered species. The lizards were collected by hand and then kept in cloth bags. The captured live lizards were kept temporarily at the Faculty of Biology at Havana University, then exported to Japan and kept and bred at Tohoku University. Exportation permits were provided by the Center of Environmental Control of Cuba (Authorized Signature by Jorge Alvarez Alvarez). Some females of *A*. *sagrei* were imported from the United States through a Japanese pet shop company (Samurai Japan Replies Co.) in 2013 (for the isolation of tissue extracts). *A*. *sagrei* individuals found in the United States had become established from Cuba, so they were actually the same species as those from Cuba. They were kept in cages at approximately 28°C with humidity above 60% for 1 month prior to experiments. The lizards were sedated and then sacrificed by decapitation. All of animal care and breeding procedures were performed in accordance with the guidelines of the Animal Care and Use Committee of Tohoku University. The protocol was approved by the committee (permit number: 19SeiShi-6 and 2011SeiDou-26). The permit to keep the species as an invasive exotic species in captivity in Japan (permit number: 06002353) were provided by the Ministry of the Environment, Japan. To provide heat shock treatment, the rearing cages were placed in an incubator at 42°C for 1 h. Western clawed frogs, *Xenopus tropicalis*, which were provided by the Institute of Amphibian Biology, Hiroshima University, through the National Bio-Resource Project of MEXT, Japan, were kept in cages at approximately 25°C. To provide heat shock treatment, the rearing cages were placed in a water bath at 31°C or 33°C for 1 h. Experimental protocols relating to these frogs were reviewed by the Committee for Ethics on Animal Experiments of Yamaguchi University Graduate School of Medicine (permit number: 06–003).

### Molecular cloning of lizard and frog HSFs

We amplified lizard HSF cDNAs by RT-PCR with LA Taq polymerase (Takara Biosciences, Kyoto) using total RNA isolated from the hindlimb and eye of *A*. *sagrei*, and cloned them into a pCR2.1-TOPO vector (Invitrogen). The PCR primers shown in S1 Table were designed based on RNA sequence data from *A*. *sagrei* [30] and Ensembl genome sequence data from *Anolis carolinensis* (http://www.ensembl.org/index.html). The sequences of multiple cDNA clones from each *HSF* gene were verified using a BigDye Terminator v3.1 Cycle Sequencing Kit and 3500 Genetic Analyzer (Applied Biosystems), and one representative full-length cDNA clone per gene (pTOPO2.1-AsHSF1 to pTOPO2.1-AsHSF4) was selected for analysis. We also amplified frog HSF cDNAs by RT-PCR using total RNA isolated from stage 39 embryos of *X*. *tropicalis*, and cloned them into a pcDNA3.1 vector (Invitrogen) at the EcoRI/XhoI sites. The PCR primers were designed based on Ensembl genome sequence data from *X*. *tropicalis* (S1 Table). After sequencing multiple cDNA clones, one representative full-length cDNA clone per gene (pcDNA3.1-XtHSF1 to pcDNA3.1-XtHSF4) was selected for analysis. Predicted amino acid sequences of HSFs from various vertebrate species were compared using GENETYX-MAC software (Software Development Co., Ltd., Tokyo). The DDBJ accession numbers for AsHSF1, AsHSF2, AsHSF3, and AsHSF4 are LC198691, LC198692, LC198693, and LC198694, respectively.

### Generation of adenoviral expression vectors

Adenoviral vectors expressing chicken and mouse HSF3 (Ad-cHSF3 and Ad-mHSF3) were generated previously [14]. An expression vector encoding hemagglutinin (HA)-tagged AsHSF1 at the C terminus (AsHSF1-HA) was created using a PCR-mediated method [14]. The cDNAs amplified by PCR were digested with BamHI and XhoI and inserted into a pcDNA3.1 vector (Invitrogen) at the BamHI/XhoI sites (pcDNA3.1-AsHSF1-HA). cDNAs for AsHSF2-HA, AsHSF3-HA, and AsHSF4-HA were also amplified by PCR and inserted into a pcDNA3.1 vector at the SalI/NotI sites (pcDNA3.1-AsHSF2-HA to pcDNA3.1-AsHSF4-HA). The KpnI/XhoI fragments of pcDNA3.1-AsHSF1-HA and the others were then inserted into a pShuttle-CMV vector (Stratagene) at the KpnI/XhoI sites (pShuttle-CMV-AsHSF1-HA to pShuttle-CMV-AsHSF4-HA). pShuttle-CMV expression vectors encoding HA-tagged XtHSF1, XtHSF2, XtHSF3, or XtHSF4 at the C-terminus were also created using a PCR-mediated method. The KpnI/XhoI fragment of each cDNA was inserted into a pShuttle-CMV vector at the KpnI/XhoI sites (pShuttle-CMV-XtHSF1-HA to pShuttle-CMV-XtHSF4-HA). Viral DNA containing the cDNA for each AsHSF was generated in accordance with the manufacturer’s instructions for an AdEasy adenoviral vector system (Stratagene). Viruses were infected into HEK293 cells, and the virus particles were enriched by CsCl gradient centrifugation and stored at -80^°^C until use.

### Adenoviral infection

To overexpress each HSF, HSF1-/- MEF cells (stock #4) were infected with an adenovirus expressing each HSF (5 x 10^7^ pfu/ml) for 2 h, and maintained with normal medium for 46 h.

### Generation of antisera

To generate bacterial expression vectors for GST fusion proteins, partial cDNA fragments encoding AsHSF3 (amino acids 300–453) and XtHSF3 (amino acids 300–553) were inserted into a pGEX-2T vector (GE Healthcare). Recombinant GST fusion proteins were expressed in *Escherichia coli* by incubating with 0.4 mM isopropyl β-D-1- thiogalactopyranoside (IPTG) at 37°C for 3 h. Bacterial lysates were separated by sodium dodecyl sulfate (SDS)-polyacrylamide gel electrophoresis (PAGE), and the respective fusion proteins were excised, electroeluted, and concentrated using Amicon ultra-4 centrifugal filter units (EMD Millipore). The proteins were emulsified with an equal volume of TiterMax adjuvant (Sigma-Aldrich) to immunize rabbits. Blood samples were collected from rabbit marginal ear veins using 18-gauge needles, and were allowed to clot for 60 min at 37°C. The clots were placed at 4°C overnight to allow them to contract. The antisera for AsHSF1 (anti-AsHSF3-1) and XtHSF3 (anti-XtHSF3-2) were extracted from the clots by centrifugation at 10,000 g for 10 min at 4°C, and were stored at -80°C after the addition of sodium azide to 0.02%.

### Western blotting

Cells were lysed in NP-40 lysis buffer {150 mM NaCl, 1.0% Nonidet P-40, 50 mM Tris (pH 8.0), 1 mM phenylmethylsulfonyl fluoride, 1 μg/ml leupeptin, and 1 μg/ml pepstatin}, and centrifuged at 12,000 x g for 10 min. Aliquots of protein (40 μg to 160 μg) were subjected to SDS-PAGE and the transferred onto nitrocellulose membranes. The membranes were blotted using rabbit antisera for HSF1 (anti-cHSF1x) [31], HSF2 (anti-mHSF2-4) [18], mouse HSF3 (anti-mHSF3-1) [14], chicken HSF3 (anti-cHSF3γ) [24], lizard HSF3 (anti-AsHSF3-1) (see above), frog HSF3 (anti-XtHSF3-2) (see above), chicken HSP70 (anti-cHSP70a) [32], mouse HSP60 (anti-mHSP60-1), and human HSP40 (anti-hHSP40-1) [31], and mouse antibodies for HSP70 (W27, Santa Cruz), β-actin (AC-15, Sigma) and GFP (GF200, Nacalai). Peroxidase conjugated goat anti-rabbit IgG and anti-mouse IgG (Cappel) were used as second antibodies. Signals were detected on X-ray films (Super RX, Fujifilm) using Amersham ECL start Western blotting detection reagent (GE Healthcare).

### Gel filtration

Whole cell extracts (200 μl containing 500 to 1,000 μg of protein) of control or heat-shocked cells were applied on a Superdex 200 10/300 GL chromatographic separation column with an AKTA fast protein liquid chromatography apparatus (GE Healthcare). The samples were eluted at 0.3 ml/min with a buffer containing 1% glycerol, 20 mM Tris-HCl (pH 7.9), 200 mM KCl, and 1.5 mM MgCl_2_. The fractions (0.5 ml) were precipitated with trichloroacetic acid (10%, final concentration), washed with acetone, dried, suspended in gel sample buffer, and analyzed by SDS-PAGE and Western blotting. The protein standards were as follows: thyroglobulin, 669 kDa; ferritin, 440 kDa; aldolase, 158 kDa; albumin, 67 kDa (GE Healthcare).

### Electrophoretic mobility shift assay

Whole cell extracts were prepared in buffer C (20 mM HEPES, pH7.9, 25% glycerol, 0.42 M NaCl, 1.5 mM MgCl_2_, 0.2 mM EDTA, 0.5 mM PMSF, and 0.5 mM DTT), and aliquots of the extracts (10 μg proteins) were subjected to electrophoretic mobility shift assays (EMSAs) using a ^32^P-labelled ideal HSE oligonucleotide as described previously [21]. Tissues from lizards and frogs were also lysed in buffer C using pestles for 1.5 ml microcentrifuge tubes. To perform antibody supershift experiments, 2.0 ml of diluted antiserum (1:10 or 1:2.5 dilution in PBS) and cell lysates (10 μg proteins) in a total volume of 10 μl were incubated on ice for 20 min. They were then mixed with a binding mixture containing an oligonucleotide probe for 20 min at room temperature, and analyzed on 4% native polyacrylamide gels [21].

### Assessment of mRNA

Total RNA was isolated from the cells using TRIzol (Ambion), and first-strand cDNA was synthesized using avian myeloblastosis virus reverse transcriptase (AMV-RT) and oligo (dT)_20_ in accordance with the manufacturer’s instructions (Invitrogen). Real-time quantitative PCR (qPCR) was performed using StepOnePlus (Applied Biosystems) with the Power SYBR Green PCR Master Mix (Applied Biosystems) using primers for *HSP70* and *β-actin* genes as described previously [25]. All reactions were performed in triplicate with samples derived from three experiments.

### RNA interference

To knockdown *G*. *gecko HSF3 (GgeHSF3*; we use this abbreviation to distinguish it from chicken *Gallus gallus* HSF3) and *HSF1* (*GgeHSF1*) genes in GL-1 cells, these cDNA sequences were determined. We first amplified a partial GgeHSF3 cDNA from GL-1 cells by RT-PCR using internal primers, and then isolated the 3’-end using a kit for 3’-rapid amplification of cDNA ends (RACE) (TAKARA Bio. Inc.). shRNA target sequences for *GgeHSF3* were determined, and corresponding sense and antisense oligonucleotides were inserted into pCR2.1-hU6 [14]. Viral DNAs and viruses including Ad-sh-GgeHSF3-KD1 and Ad-sh-GgeHSF3-KD2 were generated as described previously [14]. Similarly, partial GgeHSF1 cDNAs were isolated using primers, GgeHSF1-F1, GgeHSF1-R1 and GgeHSF1-F2, and viruses including Ad-sh-GgeHSF1-KD1 and Ad-sh-GgeHSF1-KD2 were generated. The DDBJ accession numbers for GgeHSF1 and GgeHSF3 are LC198689 and LC198690, respectively.

GL-1 cells were infected with an adenovirus expressing each shRNA (4 x 10^8^ pfu/ml) for 2 h and then maintained in normal medium for 96 h. The cells were heat shocked at 42^°^C for 1 h and allowed to recover at 30^°^C for 3 or 6 h.

### Gene disruption using the CRISPR/Cas9 system

gRNAs targeting the *GgeHSF3* gene were designed using an online tool developed by Dr. Feng Zhang (http://crispr.mit.edu/). The DNA sequences including gRNAs of the *GgeHSF3* gene were cloned into an pX330 vector (Addgene, catalog no. 42230) at the BbsI site [33]. The DNA sequences were: gk3-455F-F (for clone 303), 5’-cac cGG AAG GAG GTG GCG TCT CTG-3’; gk3-455F-R (for clone 303), 5’-aaa cCA GAG ACG CCA CCT CCT TCC-3’; gk3-6F-F (for clone 109), 5’- cac cGG GCT TCC TGG CCA AGC TCT-3’; gk3-6F-R (for clone 109), 5’-aaa cAG AGC TTG GCC AGG AAG CCC-3’ (the gRNA-encoding sequences are indicated by capital letters). pX330-GgeHSF3-1 or pX330-GgeHSF3-2 was electroporated into GL-1 cells with the use of Amaxa MEF2 Nucleofector Kit (Lonza, VAPD-1005) using a Nucleofector 2b device (Lonza, program A-023), and the cells were then maintained in Ham's F-12K containing 15% FBS. Cell extracts were prepared from all of the colonies and subjected to Western blotting using anti-HSF3 antibody. To confirm the deletion or insertion of the *GgeHSF3* gene, partial cDNAs were amplified by RT-PCR, and then inserted into a pCR2.1-TOPO vector (Invitrogen). In HSF3-null GL-1 clone 303, two copies of the *GgeHSF3* gene contained a deletion of two nucleotides (CT) and insertion of one nucleotide (T), respectively, at putative cleavage site 1. In clone 109, two nucleotides (CT) were deleted at putative cleavage site 2 in two copies of the *GgeHSF3* gene.

### Statistical analysis

Data were analyzed using Student’s t-test or analysis of variance (ANOVA). Asterisks in figures indicate that differences were significant (*P* < 0.05 or 0.01). Error bars represent the standard deviation (s.d.) for more than three independent experiments.

## Results

### Cloning and general features of lizard and frog *HSF* genes

To isolate lizard *HSF* cDNA clones, we used the brown anole lizard, *A*. *sagrei*, which has been widely studied in terms of reptile evolutionary and thermal biology [34]. We performed RT-PCR using primers that were mainly designed on the basis of RNA sequence data from *A*. *sagrei* [30], and we isolated *HSF1*, *HSF2*, and *HSF3* cDNA clones from the hindlimb, and *HSF4* cDNA clones from the eye [13]. Frog cDNA clones for HSF1 and HSF2 were isolated previously from the African clawed frog, *Xenopus laevis*, which has a tetraploid genome [35, 36]. Therefore, we isolated frog cDNA clones for all four *HSF* genes from stage 39 embryos of the Western clawed frog, *X*. *tropicalis* (or *Silurana tropicalis*), which has a diploid genome, using primers designed on the basis of Ensembl genome sequence data of the same species (http://www.ensembl.org/index.html).

**Fig 1:**
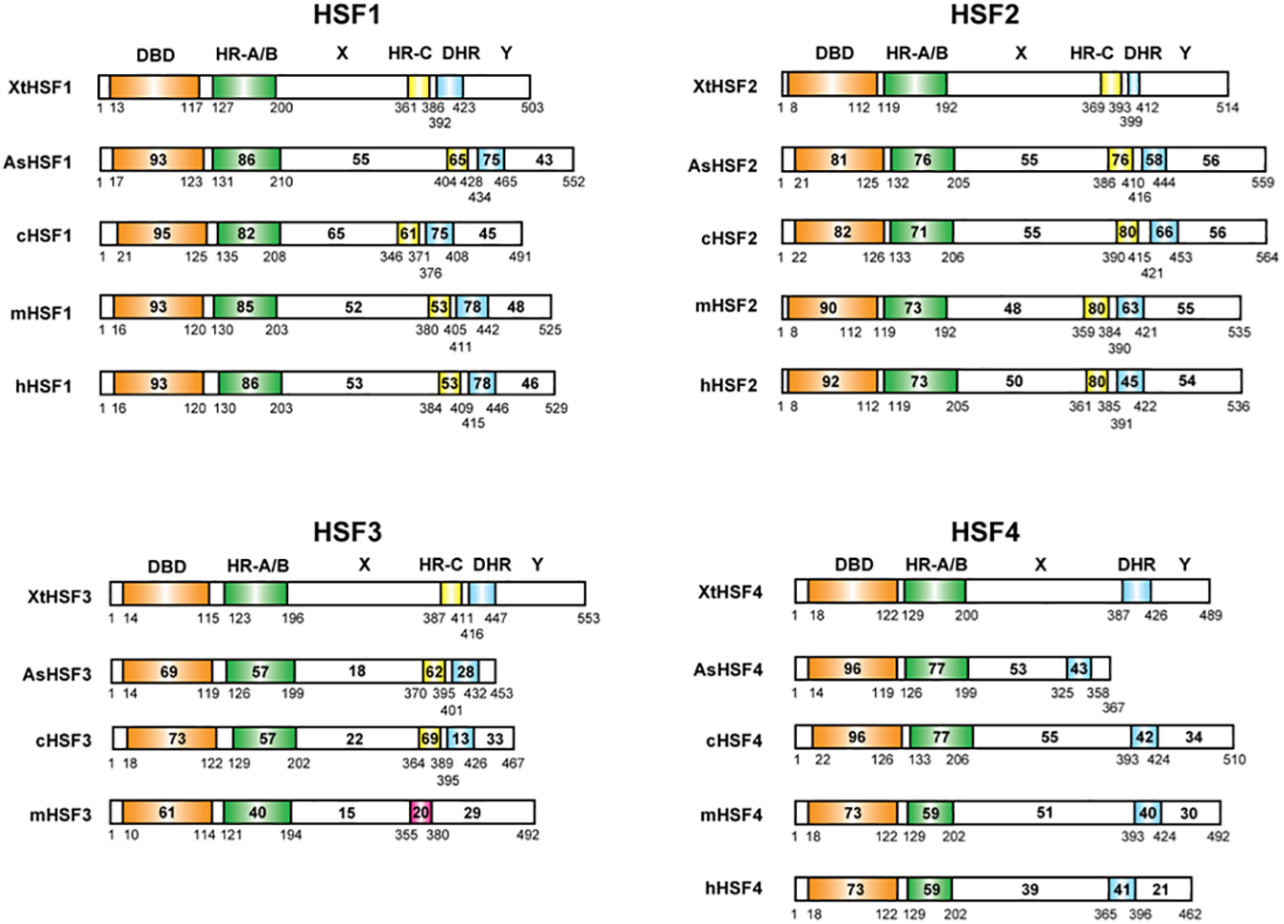
Diagrammatic representation of vertebrate HSF family members. The percent identity between XtHSF1 and each HSF was established. The number of amino acids of each HSF is shown at the amino-terminal end. DBD, DNA-binding domain; HR, hydrophobic heptad repeat; DHR, downstream of HR-C. The red box in mHSF3 indicates an HR-C-like domain, in which hydrophobic amino acids are not well conserved. AsHSF and XtHSF members were identified in this study. cHSF1, cHSF2, and cHSF3 [21]; cHSF4 [14]; mHSF1 and mHSF2 [48]; mHSF3 [14]; mHSF4 [38]; hHSF1 [39]; hHSF2 [40]; hHSF4 [41].

The predicted amino acid sequences of lizard (*A*. *sagrei*) HSFs (AsHSFs) and frog (*X*. *tropicalis*) HSFs (XtHSFs) were compared with those of chicken (*Gallus gallus*) [14, 21], mouse (*Mus musculus*) [14, 37, 38], and human (*Homo sapiens*) [39–41] HSFs (cHSFs, mHSFs, and hHSFs, respectively) using the computer program GENETYX-MAC. AsHSFs and XtHSFs possess an N-terminal DNA-binding domain (DBD) and a neighboring oligomerization domain that consists of hydrophobic heptad repeats (HR-A/B) (Fig 1) [42–44]. Except for HSF4, other HSFs also have hydrophobic heptad repeat (HR-C) near the C-terminals, which inhibits the oligomerization of HR-A/B [45, 46]. There is also a variant of the hydrophobic heptad repeat, a downstream of HR-C (DHR) [41]. The sequences of regions X and Y in HSF1s and HSF2s as well as those of region X in HSF4s are highly conserved among vertebrate species, whereas the sequences in these two regions in HSF3s are less well conserved (Fig 1).

### Relationship between vertebrate HSF family members

**Fig 2:**
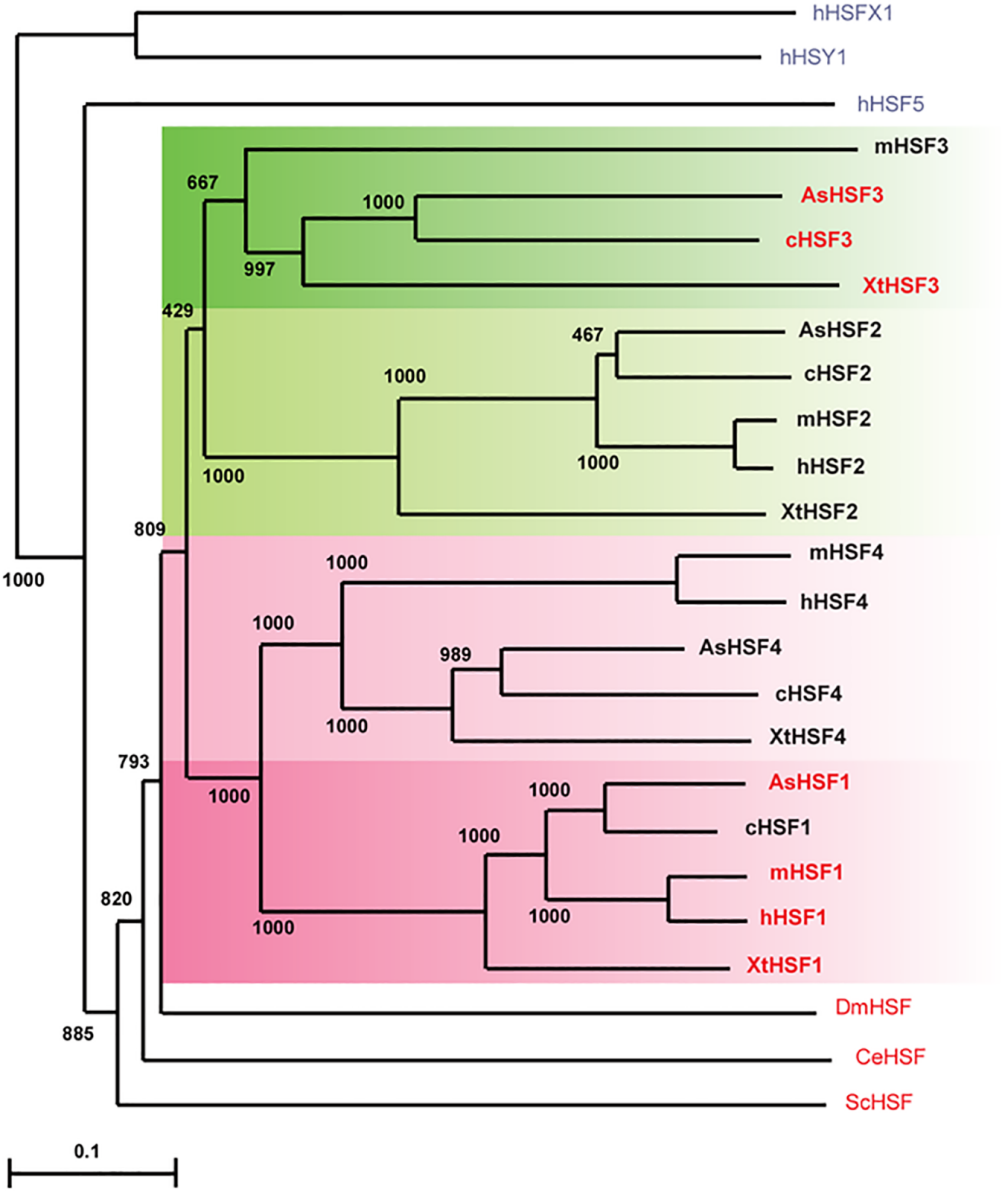
Phylogenetic tree of vertebrate HSF members. The phylogenetic tree was generated in CLUSTAL W [62]. Gaps were excluded from all phylogenetic analyses. The numbers represent bootstrap values (1,000 bootstrap replicates were performed). The unrooted tree was drawn using the program TREEVIEW [63]. Bar represents 0.05 substitutions per site. Amino acid sequences used in tree construction are those of non-vertebrate HSFs from yeast *Saccharomyces cerevisiae* (ScHSF), *Drosophila melanogaster* (DmHSF), and *Caenorhabditis elegans* (CeHSF), and the human HSF superfamily members, hHSFX1, hHSFY1, and hHSF5. Accession numbers of these amino acid sequences were shown previously [14, 52]. HSFs that have a potential to induce HSP expression during heat shock are indicated in red (see the results in this study).

We previously constructed the phylogenetic tree from the predicted full-length amino acid sequences of chicken, mouse, and human HSFs. The resulting tree suggested that HSF1 is most closely related to HSF4, and the nucleic acid sequences of HSF3 have changed quickly through time [14]. Here, we reconstructed a robust phylogenetic tree of vertebrate HSFs, including lizard and frog HSFs (Fig 2). This phylogenetic tree confirmed that the HSF1 and HSF4 clusters are clearly separated from the HSF2 and HSF3 clusters. Furthermore, HSF3 in one species was unrelated to that in other species among HSF family members. The latter observation was validated by comparison of amino acid sequence identities in the DBD and HR-A/B domains of vertebrate HSFs (Fig 1). This finding supports the notion that the *HSF3* gene had diverged most quickly during evolution. In addition, the phylogenetic relationships of HSF4s among species were unique. Human and mouse HSF4s were highly related, but other HSF4s were only distantly related to mammalian HSF4s (Fig 2). This observation may imply that non-mammalian *HSF4* genes have diverged differently from mammalian *HSF4* genes during evolution [47].

### Heat shock induces HSE-binding activities of HSF1 and HSF3 in lizards and frogs

**Fig 3:**
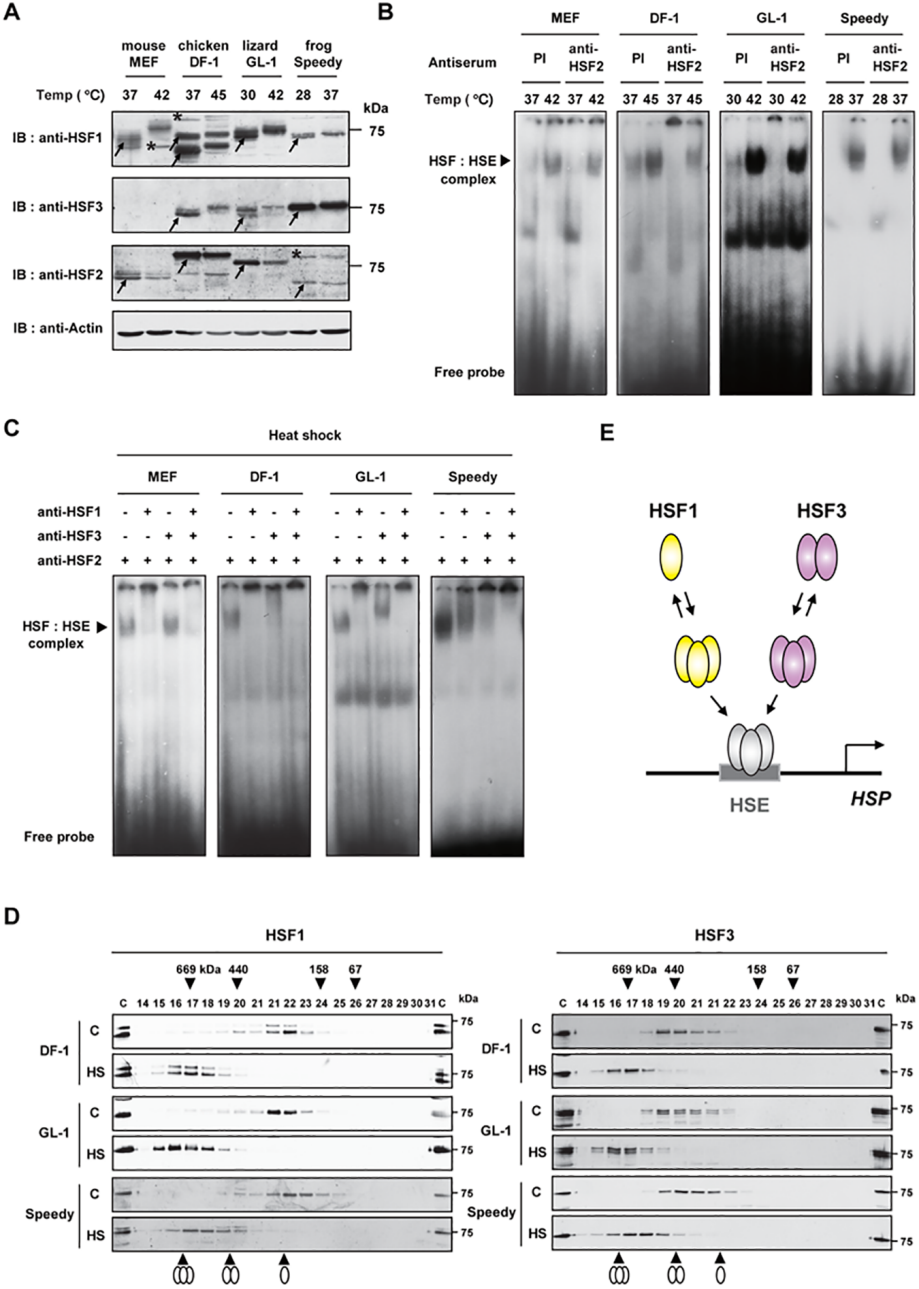
Heat shock induces the HSE-binding activity of HSF1 and HSF3 in lizard and frog cells. (**A**) Expression of HSF1 and HSF3 in mouse (MEF), chicken (DF-1), lizard (GL-1), and frog (Speedy) cells. MEF and DF-1 cells maintained at 37^°^C were heat shocked for 1 h at 42^°^C and 45^°^C, respectively. GL-1 cells maintained at 30 and Speedy cells maintained at 28^°^C were heat shocked for 1 h at 42 and 37^°^C, respectively. Cell extracts (48 μg protein per sample) were subjected to western blotting using anti-HSF1 (anti-cHSF1x), anti-HSF3 (anti-XtHSF3-2), anti-HSF2 (anti-mHSF2-4), or anti-β-actin antibodies. Arrows indicate specific bands of HSF1 in MEF (70 kDa), DF-1 (72 and 65 kDa), GL-1 (72 kDa), and Speedy (72 kDa) cells, those of HSF2 in MEF (70 kDa), DF-1 (80 kDa), GL-1 (74 kDa), and Speedy (68 kDa) cells, and those of HSF3 in DF-1 (75 kDa), GL-1 (74 kDa), and Speedy (76 kDa) cells in unstressed conditions. Stars indicate non-specific bands. (**B**) Induction of the HSE-binding activity in mouse, chicken, lizard, and frog cells during heat shock. Cells were heat shocked as described in A. Whole cell extracts were prepared and aliquots (10 μg proteins) were subjected to EMSA in the presence of 2 μl of 1:10-diluted preimmune (PI) or anti-HSF2 (anti-mHSF2-4) serum. (**C**) Analysis of heat-induced HSE-binding activity. Whole cell extracts from heat-shocked cells described in B (10 μg proteins) were subjected to antibody supershift experiments using anti-HSF1 (anti-cHSF1γ) or anti-HSF3 (anti-mHSF3-1, anti-cHSF3γ, anti-AsHSF3-1, or anti-XtHSF3-2) antibodies in the presence of anti-HSF2 (anti-mHSF2-4) antibody. (**D**) Gel filtration analysis of HSF1 and HSF3. Whole cell extracts described in B were subjected to gel filtration [24]. Proteins were subjected to western blotting using anti-HSF1 (anti-cHSF1x) or anti-HSF3 (anti-XtHSF3-2) antibodies. The elution positions of monomers, trimers, and dimers are shown at the bottom. (**E**) Oligomeric states of HSF1 and HSF3 in lizard and frogs. HSF1 stayed mostly as an inert monomer in unstressed condition, whereas HSF3 was an inert dimer. Upon heat shock, both HSF1 and HSF3 converted to active trimers that bind to the HSE.

To elucidate the roles of HSF1 and HSF3 in the heat shock response, we first investigated the expression of HSF1 and HSF3 proteins by western blotting. An anti-cHSF1x antibody, which was raised against a full-length cHSF1 [31], recognized lizard and frog HSF1 as well as human and chicken HSF1 (see below), but an anti-cHSF3γ antibody, which was raised against the C-terminus of cHSF3 (amino acids 391–467) [24], did not recognized lizard or frog HSF3 (S1 Fig, panel A). Therefore, we generated an anti-AsHSF3-1 antibody raised against the C-terminus of AsHSF3 (amino acids 300–453) and an anti-XtHSF3-2 antibody raised against the C-terminus of XtHSF3 (amino acids 300–553), and then we determined whether they recognized various HSF3 proteins overexpressed in MEF cells. We found that the anti-XtHSF3-2 antibody recognized frog, lizard, and chicken HSF3, whereas the anti-AsHSF3-1 antibody only recognized lizard and chicken HSF3 (S1 Fig, panel A). By using anti-cHSF1x and anti-XtHSF3-2 antibodies, we found that the HSF1 and HSF3 proteins are expressed in lizard GL-1 (derived from *G*. *gecko*) [27] and frog Speedy (derived from *X*. *tropicalis*) [28] cells (Fig 3A). Lizard HSF1 and HSF3 bands as well as frog HSF1 bands were composed of multiple bands, and their mobility was retarded in heat shocked cells, which was similar to chicken HSF1 and HSF3. In contrast, frog HSF3 was detected as a single band, and heat shock hardly affected its mobility. Western blotting with an anti-mHSF2-4 antibody, which was raised against mHSF2 lacking the N-terminal DNA-binding domain (amino acids 107–517) [18], recognized cHSF2, AsHSF2, and XtHSF2 (S1 Fig, panel B) and thereby showed the expression of HSF2 in lizard and frog cells (Fig 3A).

It was shown previously that heat shock-induced HSF:HSE complexes is mostly composed of HSF1 in mouse cells [48, 49], whereas those are composed of both HSF1 and HSF3 in avian cells [24]. To examine the HSE-binding activity of HSF1 and HSF3, we determined the specificity of each antibody using an electrophoretic mobility shift assay (EMSA) and antibody supershift experiments with extracts from heat shocked HSF1-null MEF cells, in which XtHSF1 or XtHSF3 was overexpressed. Overexpressed XtHSF1 and XtHSF3 bound to the HSE in both control and heat-shocked cells (S1 Fig, panel C, lanes 1, 2, 9, 10). An anti-cHSF1γ antibody, which was raised against the C-terminus of cHSF1 (amino acids 373–491) [50], supershifted the XtHSF1:HSE complex (lanes 5, 6), but it was unable to affect the XtHSF3:HSE complex (lanes 13, 14). Conversely, an anti-XtHSF3-2 antibody supershifted only the XtHSF3:HSE complex (lanes 7, 8, 15, 16). We examined the HSE-binding activity of control and heat-shocked cells in the presence of preimmune serum, or an anti-mHSF2-4 antibody to remove the HSE-binding activity of HSF2 [50]. We found that the HSE-binding activity was induced during heat shock in lizard GL-1 and frog Speedy cells as well as mouse and chicken cells, and the induced HSE-binding activity was mostly composed of HSFs other than HSF2 (Fig 3B). In contrast, the constitutive HSE-binding activities at least in DF-1 and GL-1 cells were detected clearly and partially supershifted by anti-HSF2 antibody. Antibody supershift experiment using anti-HSF1 or anti-HSF3 antibody in the presence of anti-HSF2 antibody showed that both HSF1 and HSF3 were components of the HSF:HSE complex in extracts of heat-shocked lizard and frog cells (Fig 3C), like in heat-shocked avian cells [24]. We then examined the oligomeric states of HSF1 and HSF3 by gel filtration, and found that HSF1 exists as a monomer in GL-1 and Speedy cells as well as chicken DF-1 cells in control conditions, whereas HSF3 exists as a dimer (Fig 3D). All of them were converted to trimers during heat shock. Thus, HSF1 undergoes a monomer-to-trimer transition during heat shock, whereas HSF3 undergoes a dimer-to-trimer transition (Fig 3E). Furthermore, we found that HSF3 is co-precipitated with HSF1 when they are overexpressed in cells (S2 Fig). This result suggested that HSF1 and HSF3 cooperate with each other, although composition of the complex is unclear.

**Fig 4:**
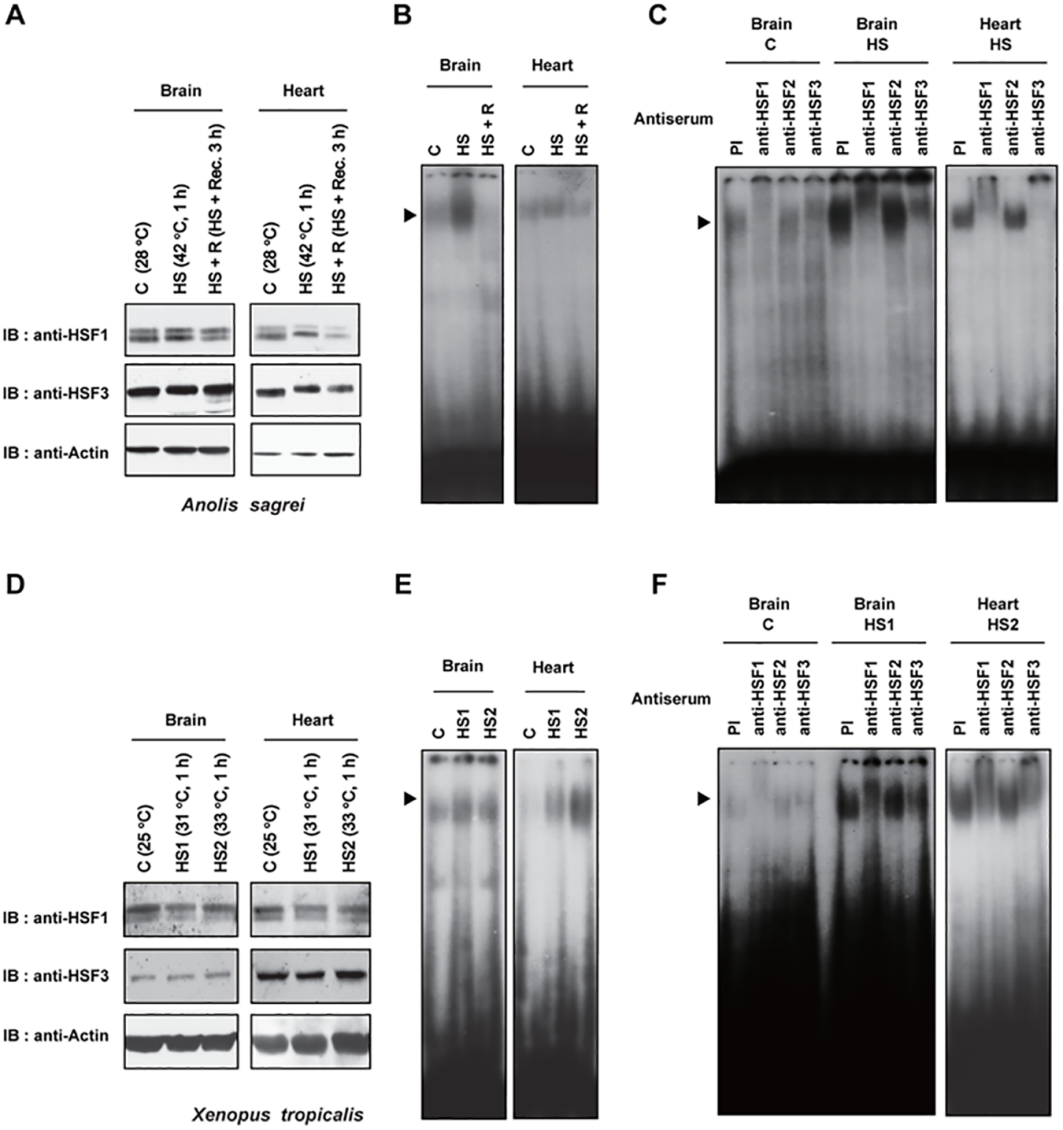
Heat shock induces the HSE-binding activity of HSF1 and HSF3 in lizard and frog tissues. (**A**) Expression of HSF1 and HSF3 in lizard tissues. Whole cell extracts were prepared from the brain and heart of control lizard *Anolis sagrei* maintained at 28^°^C (C), lizards heat-shocked at 42^°^C for 1 h (HS), or the lizards that were heat-shocked and then recovered at 28^°^C for 3 h. Aliquots (80 μg proteins) were subjected to western blotting using anti-HSF1 (anti-cHSF1x), anti-HSF3 (anti-AsHSF3-1), or anti-β-actin antibodies. (**B**) Induction of the HSE-binding activity in lizard tissues during heat shock. Aliquots (20 μg proteins) of whole cell extracts described in A were subjected to EMSA. An arrowhead indicates the HSF:HSE complex. (**C**) Analysis of heat-induced HSE-binding activity. Whole cell extracts from the tissues described in A (20 μg proteins) were subjected to antibody supershift experiments using anti-HSF1 (anti-cHSF1γ), anti-HSF2 (anti-mHSF2-4), or anti-HSF3 (anti-AsHSF3-1) antibodies, or preimmune (PI) serum. Each 1:10-diluted antiserum (2 μl) was added in the binding mixture. (**D**) Expression of HSF1 and HSF3 in frog tissues. Whole cell extracts were prepared from the brain and heart of control frog *Xenopus tropicalis* maintained at 25^°^C (C), the lizard heat-shocked at 31^°^C for 1 h (HS1) or at 33^°^C for 1 h (HS2). Aliquots (80 μg proteins) were subjected to western blotting using anti-HSF1 (anti-cHSF1x), anti-HSF3 (anti-XtHSF3-2), or anti-β-actin antibodies. (**E**) Induction of HSE-binding activity in frog tissues during heat shock. Aliquots (20 μg proteins) of whole cell extracts described in D were subjected to EMSA. (**F**) Analysis of heat-induced HSE-binding activity. Whole cell extracts from the tissues described in D (20 μg proteins) were subjected to antibody supershift experiments using anti-HSF1 (anti-cHSF1γ), anti-HSF2 (anti-mHSF2-4), or anti-HSF3 (anti-XtHSF3-2) antibodies, or preimmune (PI) serum.

We next examined the expression and HSE-binding activity of HSF1 and HSF3 in the tissues of lizard *A*. *sagrei* and frog *X*. *tropicalis*. HSF1 and HSF3 were expressed in the brain and heart of control (28^°^C) and heat-shocked (42^°^C, 1 h) lizards (Fig 4A). HSE-binding activity was detected in the brain and heart of control lizards and was induced in response to heat shock (Fig 4B). The elevated HSE-binding activity then reduced during recovery at 28^°^C. We found that the heat-induced HSE-binding activity was retarded in the presence of anti-HSF1 or anti-HSF3 serum under this heat shock condition, but not in the presence of anti-HSF2 serum (Fig 4C). The constitutive HSE-binding activity was also retarded clearly in the presence of anti-HSF1 or anti-HSF3 serum. Similarly, we detected the expression of HSF1 and HSF3 in the brain and heart of control (25^°^C) and heat-shocked (31 or 33^°^C for 1 h) frogs (Fig 4D), and found that heat-induced HSE-binding activity was retarded in the presence of anti-HSF1 or anti-HSF3 serum (Fig 4E and 4F). Taken together, these results indicated that the HSE-binding activities of both HSF1 and HSF3 are induced during heat shock in lizards and frogs.

### Lizard and frog HSF3 as well as HSF1 can induce HSP70 expression

**Fig 5:**
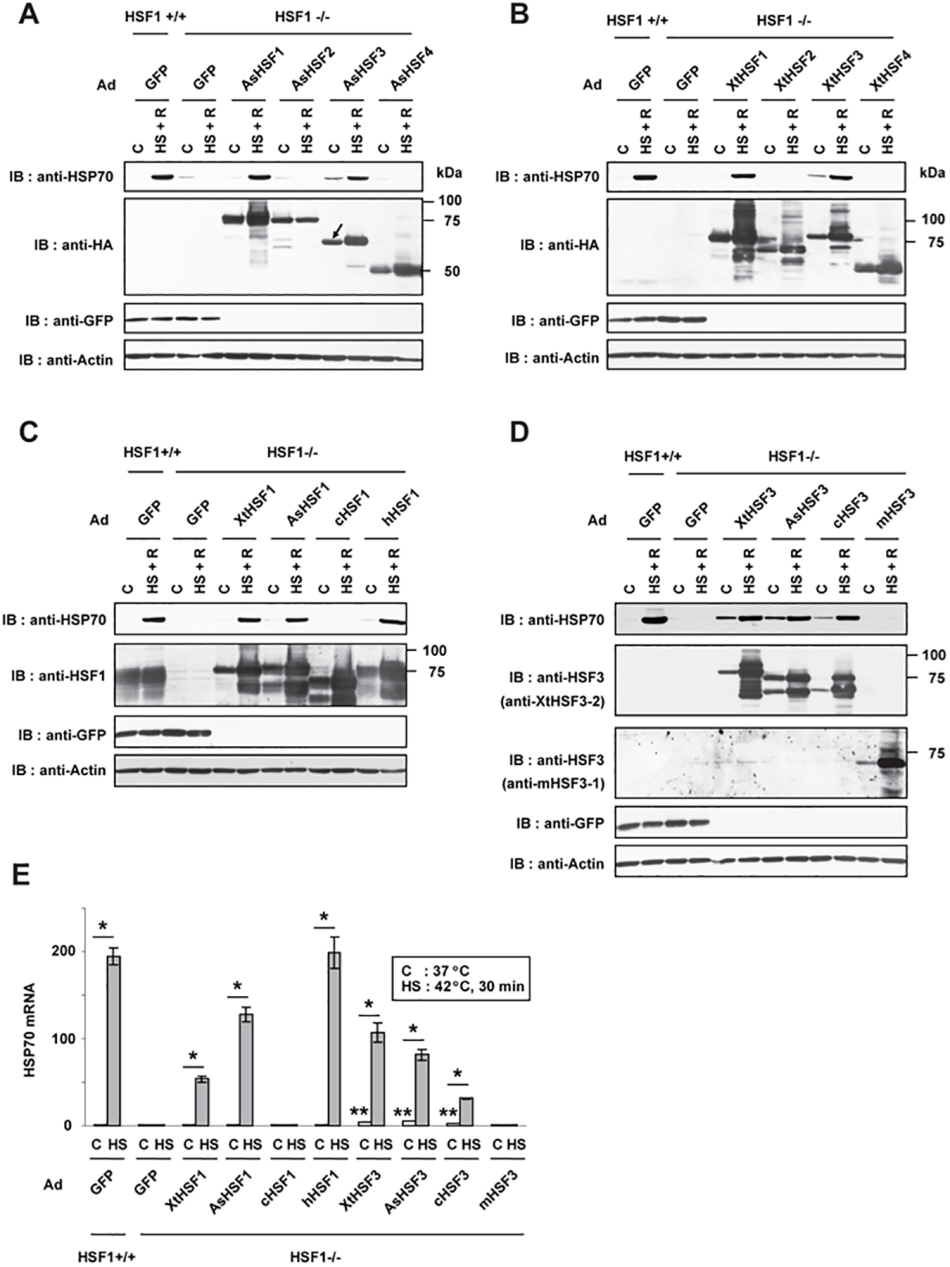
Lizard and frog HSF3 as well as HSF1 can induce HSP70 expression. (**A**) Induction of HSP70 by lizard HSFs. HSF1-null MEF cells (HSF1-/-) were infected for 48 h with adenovirus expressing GFP, AsHSF1-HA, AsHSF2-HA, AsHSF3-HA, or AsHSF4-HA. These cells and wild-type MEF cells (HSF1+/+) were untreated (C) or treated with heat shock at 42^°^C for 1 h and allowed to recover for 3 h (HS + R). Cell extracts were prepared from these cells and aliquots were subjected to western blotting using the indicated antibodies. Positions of molecular weight markers are indicated. Only the lower band of AsHSF3-HA (arrow) was detected by anti-HA antibody. (**B**) Induction of HSP70 by frog HSFs. HSF1-null cells were infected with adenovirus expressing GFP, XtHSF1-HA, XtHSF2-HA, XtHSF3-HA, or XtHSF4-HA. These cells were treated and analyzed as described in A. (**C**) Induction of HSP70 by vertebrate HSF1 members. HSF1-null cells were infected with adenovirus expressing GFP, XtHSF1-HA, AsHSF1-HA, cHSF1, or hHSF1, and were treated as described in A. Aliquots of cell extracts were subjected to western blotting using the indicated antibodies including anti-HSF1 (anti-cHSF1x). (**D**) Induction of HSP70 by vertebrate HSF3 members. HSF1-null cells were infected with adenovirus expressing GFP, XtHSF3-HA, AsHSF3-HA, cHSF3, or mHSF3, and were treated as described in A. Aliquots of cell extracts were subjected to western blotting using the indicated antibodies including anti-HSF3 (anti-XtHSF3-2 or anti-mHSF3-1). (**E**) Induction of HSP70 mRNA by vertebrate HSF1 and HSF3 members. HSF1-null cells (HSF1-/-) were infected with the indicated adenoviruses for 48 h. These cells and wild-type MEF cells (HSF1+/+) were untreated (C) or treated with heat shock at 42^°^C for 30 min (HS). HSP70 mRNA levels were quantified by RT-qPCR are showen (n = 3). *, p < 0.01 versus each control value; **, p < 0.05 versus control value of GFP-expressing cells by Student’s t-test.

HSF1, but not HSF3, induced the expression of *HSP* genes during heat shock in mice [14, 51], whereas only HSF3 induced it in avian models [38, 50, 52]. Therefore, we wondered whether one of the two HSFs has a potential to induce the expression of *HSP* genes during heat shock. Because *Drosophila* HSF can robustly induce the expression of *HSP* genes in mouse cells [52], we overexpressed lizard HSF family members in HSF1-null MEF cells and examined the expression of HSP70 during heat shock. It was revealed that lizard HSF1 and HSF3, but not HSF2 and HSF4, are able to induce the expression of HSP70 during heat shock (Fig 5A). Likewise, frog HSF1 and HSF3 overexpressed in HSF1-null MEF cells induced HSF70 expression during heat shock (Fig 5B). Frog, lizard, and human HSF1s all had a similar potential to induce HSP70 expression in MEF cells (Fig 5C), whereas frog, lizard, and chicken HSF3s had similar potentials (Fig 5D). The expression of HSP70 mRNA was also induced by these HSFs during heat shock (Fig 5E). These results demonstrate that lizard and frog HSF3 as well as HSF1 can induce HSP70 expression during heat shock. It would be worth noting that overexpressed HSF3 members slightly elevated the expressions of HSP70 even in unstressed condition (Fig 5D and 5E). This observation suggested that HSF1 activity is under tighter negative regulation than HSF3 activity in unstressed conditions.

### Both HSF3 and HSF1 induce heat shock response in lizard cells

**Fig 6:**
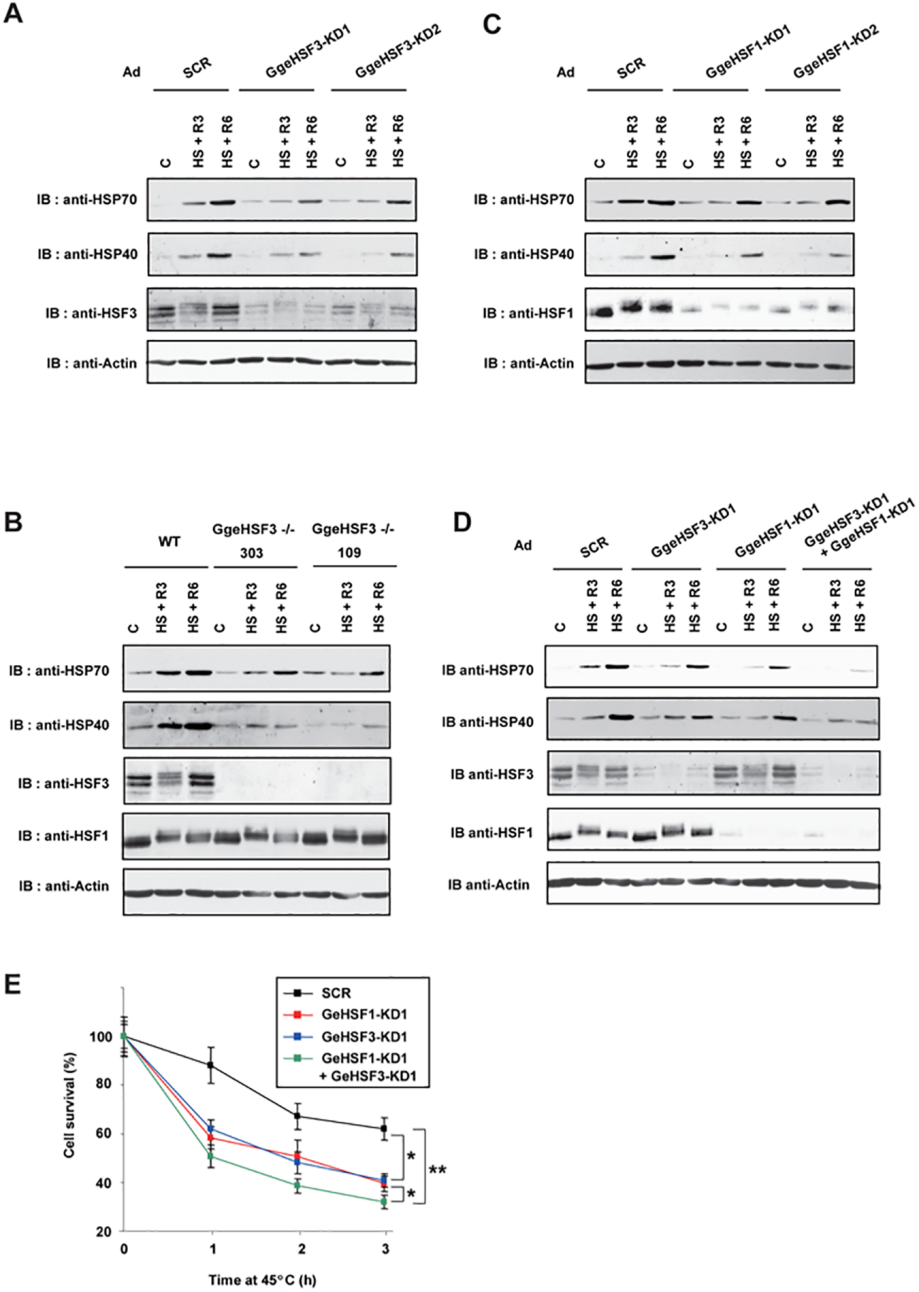
HSF1 and HSF3 induce heat shock response in lizard cells. (**A**) Induction of HSP70 in HSF3 knockdown cells. *Gekko gecko* GL-1 cells were infected with Ad-sh-SCR, Ad-sh-GgeHSF3-KD1, or Ad-sh-GgeHSF3-KD2 for 96 h, and were then heat shocked at 42^°^C for 1 h and allowed to recover for 3 h (HS + R3) or 6 h (HS + R6). Cell extracts were prepared from these cells, and aliquots were subjected to western blotting using anti-HSP70 (anti-cHSP70a), anti-HSF3 and anti-β-actin antibodies. (**B**) Induction of HSP70 in HSF3 knockout cells. Wild-type (WT) and HSF3-null GL-1 cells (HSF3-/- clones 303 and 109) were heat-shocked at 42^°^C for 1 h and allowed to recover for 3 h (HS + R3) or 6 h (HS + R6). Cell extracts were prepared and aliquots were subjected to western blotting. (**C**) Induction of HSP70 in HSF1 knockdown cells. GL-1 cells were infected with Ad-sh-SCR, Ad-sh-GgeHSF1-KD1, or Ad-sh-GgeHSF1-KD2 for 96 h, and were treated as in A. (**D**) Induction of HSP70 in HSF3 and HSF1 knockdown cells. GL-1 cells were infected with the indicated adenoviruses for 96 h, and were treated as in A. Cell extracts were prepared from these cells, and aliquots were subjected to western blotting using antibodies including anti-HSP70, anti-HSP40 (anti-hHSP40-1), and anti-HSP60 (anti-mHSP60-1) antibodies. (**E**) Cell survival under heat shock conditions. GL-1 cells were infected with Ad-sh-SCR, Ad-sh-GgeHSF1-KD1, Ad-sh-GgeHSF3-KD1, or both viruses for 96 h, and were then heat shocked at 45^°^C for the indicated periods. These cells were stained with trypan blue, and percentages of surviving cells are shown (n = 3). *, p < 0.05; **, p < 0.01 by ANOVA.

To examine whether one of two HSFs plays a dominant role in the HSR, we first isolated a partial cDNA clone for *G*. *Gecko HSF3* (*GgeHSF3*) gene from GL-1 cells and generated adenoviruses expressing short hairpin RNAs targeting for the *GgeHSF3* gene (S3 Fig, panel A). We found that HSF3 protein levels were severely reduced in GL-1 cells infected with Ad-sh-GgeHSF3-KD1 or Ad-sh-GgeHSF3-KD2 compared with those in cells infected with adenovirus expressing scrambled RNA (Ad-sh-SCR) (Fig 6A). The expression of HSP70 and HSP40 was markedly reduced in HSF3-knockdown cells compared with scrambled-RNA expressing cells. However, substantial amounts of HSPs were still induced. To exclude the possibility that the remaining HSF3 induces the HSP expression, we then disrupted the *HSF3* gene in GL-1 cells by using a CRISPR/Cas9 system [33] and generated two HSF3-null clones (clones 303 and 109) with different mutations (S3 Fig, panel A). We confirmed that disruption of the *HSF3* gene partially reduced the heat-shock induction of HSP70 and HSP40 (Fig 6B). Next, we examined HSP expression in cells infected with Ad-sh-GgeHSF1-KD1 or Ad-sh-GgeHSF1-KD2 (S3 Fig, panel B) and found that HSF1 knockdown also partially reduced HSP expression in response to heat shock (Fig 6C). Furthermore, knockdown of both HSF3 and HSF1 severely reduced the heat-shock induction of HSP70 and HSP40 (Fig 6D). Consistently, HSF3- or HSF1-knockdown cells were more sensitive to a continuous exposure to an extreme high temperature than scrambled-RNA expressing cells, and double-knockdown cells were most sensitive to it (Fig 6E). These results indicated that HSF3 and HSF1 are not redundant but cooperatively induce the heat shock response in lizard cells.

## Discussion

Vertebrates are a group of animals that includes mammals, birds, reptiles, amphibians, and fish. Among these vertebrate animals, all four HSF family members have been extensively characterized in mammals and birds [7–11, 23]. These studies revealed that HSF1 is a mammalian orthlog of the single HSF in *Drosophila*, because it undergoes a monomer-to-trimer transition and induces the *HSP* expression during heat shock like *Drosophila* HSF [44, 45, 48, 49, 51]. On the other hand, HSF3, which undergoes a dimer-to-trimer transition during heat shock, takes the place of HSF1 in birds [22, 24, 50]. Thus, the potential of HSF3 to induce *HSP* expression has been believed to be specific to birds. We wondered whether only one of the HSF family members possesses the potential to induce the HSR in all vertebrate animals including lizards and frogs. In this study, we showed that heat shock-induced HSP70 expression was regulated by both HSF1 and HSF3 in lizard and frog cells (Figs 5 and 6). These two factors were not redundant, but regulate the HSP70 expression cooperatively (Fig 6). These observations indicated that HSF3 is not only a master regulator of the avian HSR but also plays an important role in the reptile and amphibian HSR, and the HSF3-mediated mechanisms in the HSR are prevalent in vertebrate species, except mammals.

Previous studies demonstrated that HSE-binding activity is induced in embryos or oocytes of *X*. *laevis* [53–55] and in whole bodies of three lizard species [56] in response to heat shock. Antibody supershift experiments of the frog and lizard HSF:HSE complexes were conducted using antibodies against human, mouse, or frog HSF1, and the heat-induced HSF:HSE complexes were super-shifted by these HSF1 antibodies [54–56]. On the other hand, we showed previously that the heat-induced chicken HSF:HSE complex was also substantially super-shifted by anti-chicken HSF1 antibody even though the complex was composed of HSF1 and HSF3 [50]. These observations left a possibility that HSF3 is a component of the heat-induced HSF:HSE complexes in lizard and frog cell extracts. By generating antibodies against lizard and frog HSF3, we demonstrated here that the heat-induced HSF:HSE complexes were composed of both HSF1 and HSF3 in lizard *G*. *gecko* GL-1 and frog *X*. *tropicalis* Speedy cells, and in the brain and heart of lizard *A*. *sagrei* and frog *X*. *tropicalis*. (Figs 3 and 4). Taken together, these observations suggested that the HSE-binding activities of both HSF1 and HSF3 are induced during heat shock in birds, lizards, and frogs [24]. Among them, avian HSF1 uniquely lacked the potential to induce *HSP* expression (Fig 5), although its amino acid sequence is highly conserved with those of other vertebrate orthologs (Figs 1 and 2). Amino acid residues of HSF1 in avian species that are required for the induction of *HSP* expression should be determined in future.

The previous phylogenetic tree of HSF family members led us to propose a model to explain the evolution of vertebrate *HSF* genes [9, 14]. However, the available sequence information of HSFs, especially HSF3 and HSF4, was limited at that time. Here, we reconstructed a robust phylogenetic tree of vertebrate HSF family members, and confirmed that the HSF1 and HSF4 clusters were separated from the HSF2 and HSF3 clusters (Fig 2). HSF1, as well as HSF2, in one species was highly related with its orthologs in other species. In marked contrast, HSF3 in one species was clearly unrelated with that in other species. Furthermore, human and mouse HSF4 members were highly related, but its orthologs in other species were less closely related to the mammalian HSF4 members (Fig 2). Based on these observations, we again hypothesized that four *HSF* genes might be generated through two rounds of whole-genome duplication (WGD) in vertebrate cells more than 440 million years ago [57–59]. Because fish uniquely experienced a third round of WGD, their genomes have evolved differently [58–60]. It was assumed that an ancestral gene of *HSF1* and *HSF4* as well as that of *HSF2* and *HSF3* was created by the first WGD event, and then four genes were created by the second event [9]. One (HSF1 or HSF3) of the two related genes, which were created from the same ancestral gene, has preserved the potential to induce *HSP* expression, but the other (HSF4 or HSF2) does not. Intriguingly, the sequences of *HSF1* have been highly conserved during vertebrate evolution, whereas those of *HSF3* have changed quickly in each species. Thus, the accumulation of genetic mutations during evolution could be greatly different even among stress-related transcription factor family genes with the same function [61].

In this manuscript, we identified HSF3 in lizards and frogs and characterized its role in the HSR. We showed that HSF3, as well as HSF1, has roles in regulating HSP70 expression during the HSR in lizards and frogs. In particular, we demonstrated that both HSF1 and HSF3 are required for maximal induction of HSP70 and HSP40 in lizards, suggesting that they cooperate to regulate the HSR. We also proposed a hypothesis about the evolutionary origin of the four *HSF* genes in several vertebrate species.

## Supporting information

S1 Fig Specificity of antibodies against each HSF. (**A**) Specificity of anti-HSF3 antibodies examined by western blotting. HSF1-null MEF cells, which were infected with adenovirus expressing XtHSF3-HA, AsHSF3-HA, cHSF3, or mHSF3, were treated without (C) or with heat shock at 42^°^C for 1 h, and recovery at 37^°^C for 3 h (HS + R). Extracts from these cells were subjected to western blotting using anti-XtHSF3-2, anti-AsHSF3-1, anti-cHSF3γ, anti-mHSF3-1, or anti-β-actin antibodies. Arrows indicate specific bands of HSF3 in heat-shocked conditions. Positions of molecular weight markers are indicated. (**B**) Specificity of anti-HSF2 antibodies by western blotting. HSF1-null MEF cells were infected for 48 h with adenovirus expressing hHSF2, cHSF2, AsHSF2-HA, or XtHSF2-HA. Extracts from these cells were subjected to western blotting using anti-mHSF2-4, anti-cHSF2a [24], anti-HA, or anti-β-actin antibodies. Arrows indicate specific bands of overexpressed HSF2 and a star indicates non-specific bands. Endogenous HSF2 protein in MEF cells was not detected after a short exposure of the blot to film. (**C**) Specificity of anti-HSF1 and anti-HSF3 antibodies by EMSA. HSF1-null MEF cells were infected with adenovirus expressing XtHSF1 or XtHSF3 for 48 h, and treated without (HS-) or with heat shock at 42^°^C for 1 h (HS+). Whole cell extracts were prepared from these cells and subjected to antibody supershift experiments using anti-HSF1 (anti-cHSF1γ) or anti-HSF3 (anti-XtHSF3-2) at a dilution of 1: 100 or 1: 25. HSF:HSE complexes and free probes are indicated.
(TIF)

S2 Fig Interaction between HSF1 and HSF3. HSF1-/- MEF cells were infected with an adenovirus expressing AsHSF1-Flag and AsHSF3-HA, and were treated with or without heat shock at 42^°^C for 30 min. Cells were lysed with NP-40 lysis buffer and immunoprecipitation was performed as described previously [25] using anti-cHSF1x antibody. The complexes were then subjected to western blotting using the same HSF1 or HA antibodies. One percent and ten percent of cell extracts were loaded on lanes 1 and 2 (Input) in HSF3 (anti-HA antibody) and HSF1 (anti-HSF1 antibody) blots, respectively.
(TIF)

S3 Fig Schematic representation of *Gekko gecko* HSF cDNAs. (**A**) Partial cDNA for GgeHSF3. Numbers of nucleotides are indicated. Target sequences for short hairpin RNA-mediated gene knockdown (KD1 and KD2) and for genome editing-mediated gene knockout (gRNA-1 and gRNA-2) are indicated (see Materials and methods). (**B**) Partial cDNA for GgeHSF1. Numbers of nucleotides and target sequences for short hairpin RNA-mediated gene knockdown (KD1 and KD2) are indicated.
(TIF)

S1 Table Primer sequences used to amplify AsHSF and XtHSF cDNAs. (PDF)

S1 File ARRIVE Guidelines Checklist. (PDF)
