# Supplementary figures and images for: HSF1 and HSF3 cooperatively regulate the heat shock response in lizards
Source: PLoS One. 2017 Jul 7;12(7):e0180776. doi: 10.1371/journal.pone.0180776 (PMC5501597; doi:10.1371/journal.pone.0180776)

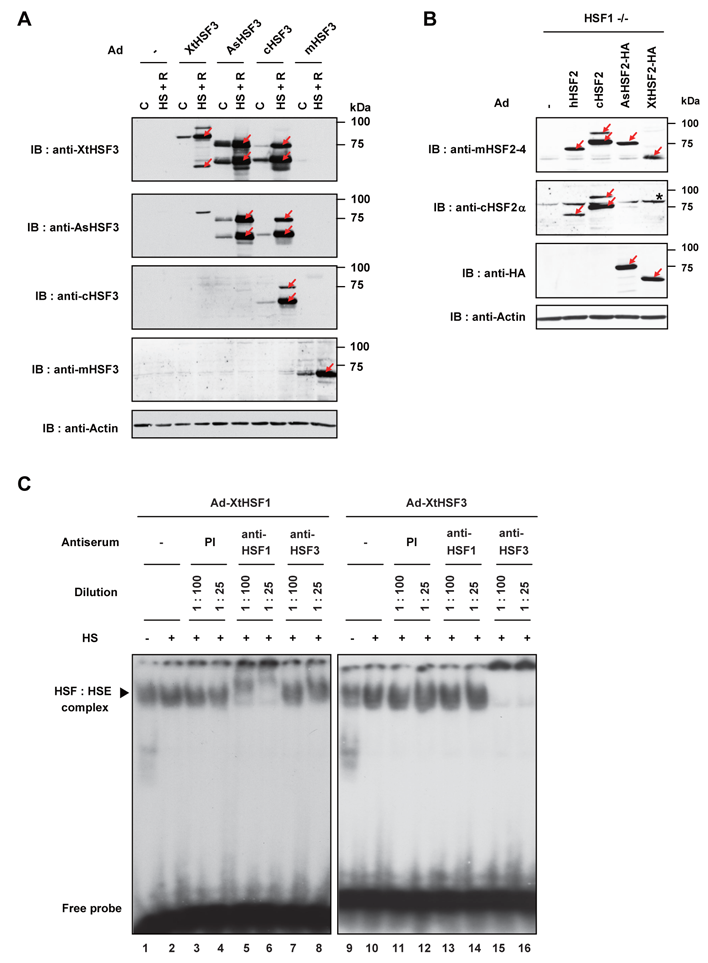

Supplement: S1 Fig — (A) Specificity of anti-HSF3 antibodies examined by western blotting. HSF1-null MEF cells, which were infected with adenovirus expressing XtHSF3-HA, AsHSF3-HA, cHSF3, or mHSF3, were treated without (C) or with heat shock at 42°C for 1 h, and recovery at 37°C for 3 h (HS + R). Extracts from these cells were subjected to western blotting using anti-XtHSF3-2, anti-AsHSF3-1, anti-cHSF3γ, anti-mHSF3-1, or anti-β-actin antibodies. Arrows indicate specific bands of HSF3 in heat-shocked conditions. Positions of molecular weight markers are indicated. (B) Specificity of anti-HSF2 antibodies by western blotting. HSF1-null MEF cells were infected for 48 h with adenovirus expressing hHSF2, cHSF2, AsHSF2-HA, or XtHSF2-HA. Extracts from these cells were subjected to western blotting using anti-mHSF2-4, anti-cHSF2a [24], anti-HA, or anti-β-actin antibodies. Arrows indicate specific bands of overexpressed HSF2 and a star indicates non-specific bands. Endogenous HSF2 protein in MEF cells was not detected after a short exposure of the blot to film. (C) Specificity of anti-HSF1 and anti-HSF3 antibodies by EMSA. HSF1-null MEF cells were infected with adenovirus expressing XtHSF1 or XtHSF3 for 48 h, and treated without (HS-) or with heat shock at 42°C for 1 h (HS+). Whole cell extracts were prepared from these cells and subjected to antibody supershift experiments using anti-HSF1 (anti-cHSF1γ) or anti-HSF3 (anti-XtHSF3-2) at a dilution of 1: 100 or 1: 25. HSF:HSE complexes and free probes are indicated. (TIF) [file pone.0180776.s001.tif]

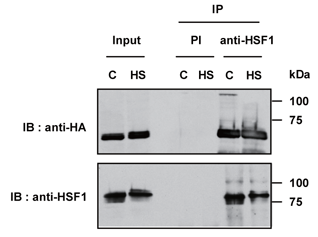

Supplement: S2 Fig — HSF1-/- MEF cells were infected with an adenovirus expressing AsHSF1-Flag and AsHSF3-HA, and were treated with or without heat shock at 42°C for 30 min. Cells were lysed with NP-40 lysis buffer and immunoprecipitation was performed as described previously [25] using anti-cHSF1x antibody. The complexes were then subjected to western blotting using the same HSF1 or HA antibodies. One percent and ten percent of cell extracts were loaded on lanes 1 and 2 (Input) in HSF3 (anti-HA antibody) and HSF1 (anti-HSF1 antibody) blots, respectively. (TIF) [file pone.0180776.s002.tif]

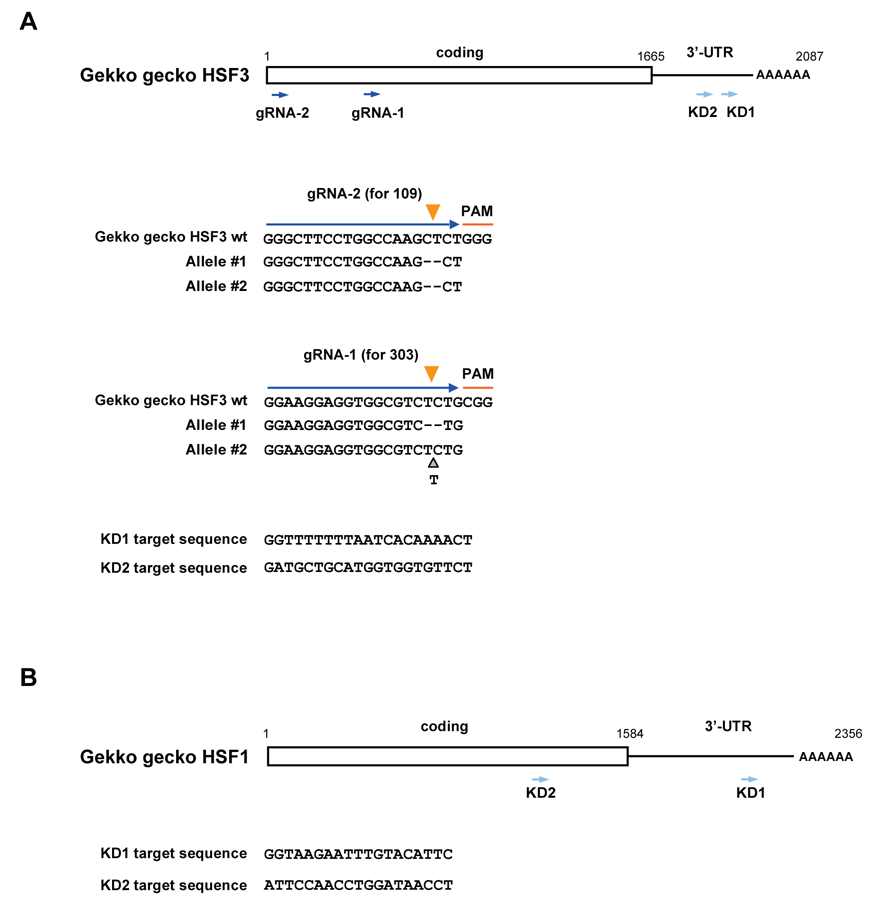

Supplement: S3 Fig — (A) Partial cDNA for GgeHSF3. Numbers of nucleotides are indicated. Target sequences for short hairpin RNA-mediated gene knockdown (KD1 and KD2) and for genome editing-mediated gene knockout (gRNA-1 and gRNA-2) are indicated (see Materials and methods). (B) Partial cDNA for GgeHSF1. Numbers of nucleotides and target sequences for short hairpin RNA-mediated gene knockdown (KD1 and KD2) are indicated. (TIF) [file pone.0180776.s003.tif]
