# Supplementary material for: HSF1 and HSF3 cooperatively regulate the heat shock response in lizards
Source: PLoS One. 2017 Jul 7;12(7):e0180776. doi: 10.1371/journal.pone.0180776 (PMC5501597; doi:10.1371/journal.pone.0180776)
Supplement: S1 Table — (PDF) [file pone.0180776.s004.pdf]

S1 Table. Primer sequences used to amplify AsHSF and XtHSF cDNAs.

	Forward Primer	Reverse Primer
AsHSF1	5'-ATGGAGGCCCCGGGCGTGG-3'	5'-CTACGAGACGGCCCCCTCCGG-3'
AsHSF2	5'-ATGAAGCAGCAGCCGGTTGC-3'	5'-TTAGTTATTTAACAATGGCATATCTGTGTCCAT-3'
AsHSF3	5'-ATGGCGGGGCGATGGCCGCGGGC-3'	5'-CATGTACTGAATCAGCTGCATGTCTG-3' (3'-UTR)
AsHSF4	5'-ATGCAGGATTCCCCCCCTTCG-3'	5'-TCATTTGCACTCTTCTCCCGATCC-3'
XtHSF1	5'-GT*GAATTC*ATGGATCCACACGGGACCTGTGGG-3'	5'-GA*CTCGAG*CTAGGAGATGCTGGAGCCCGCCGGCT-3'
XtHSF2	5'-GT*GAATTC*ATGAAACAGAACTCAAATGTTCCGGC-3'	5'-GA*CTCGAG*TTAATCCAGCAGCGGCATATCATTGTC-3'
XtHSF3	5'-GT*GAATTC*ATGGAGGAGCCGTCTGTCCCTCTGGG-3'	5'-GT*CTCGAG*TTAAAACTGTGTCTGGCTCTGTGGCTT-3'
XtHSF4	5'-GT*GGTACC*ATGCAGGAATCTGCCAGCTCCTTGGC-3'	5'-GA*CTCGAG*TCACTCGTTTTGCTCATCTGGCTGCT-3'

Red, translation start sites; Blue, translation termination sites.

Italics indicate EcoRI, KpnI or XhoI sites.
